# Point-of-care-ultrasound in cardiac arrest: a useful tool for resuscitation

**DOI:** 10.3389/fmed.2026.1795346

**Published:** 2026-03-20

**Authors:** Anandu Mathews Anto, Nismat Javed, Rahul Reddy Nallapareddy, Shivani Jani, Misbahuddin Khaja

**Affiliations:** BronxCare Health System, New York, NY, United States

**Keywords:** ACLS, asystole, cardiac arrest, critical care, PEA, POCUS

## Abstract

Cardiac arrest is defined as the sudden loss of heart activity, resulting in ineffective breathing and blood circulation. Point-of-care ultrasound (POCUS) provides real-time, evidence-based information during resuscitation and enables rapid identification of reversible conditions to guide ongoing resuscitative efforts. This mini-review focuses on the utility of POCUS during cardiac arrest, including its role in non-shockable rhythms such as pulseless electrical activity, pseudo-PEA, and asystole, where electrocardiographic findings alone may be misleading. POCUS can assist in the identification of reversible causes, including cardiac tamponade, massive pulmonary embolism, and tension pneumothorax. The review also addresses the role of serial ultrasound in monitoring cardiac activity and guiding advanced resuscitation, while emphasizing that ultrasound should not be used as the sole criterion for termination of resuscitation. We have also reviewed the practical aspects of performing POCUS during cardiac arrest, including probe selection, obtaining useful views, and strategies to minimize interruptions to chest compressions. Structured approaches such as the Cardiac Arrest Sonographic Assessment protocol are discussed to support efficient image acquisition within the 10-s pulse-check window.

## Introduction

Cardiac arrest is defined as the sudden loss of heart activity, resulting in ineffective breathing and blood circulation. It is a clinical syndrome that is confirmed by a lack of adequate circulation and can be extremely detrimental if not intervened upon. Point-of-care ultrasound (POCUS) is making waves in precision medicine by providing evidence-based care rather than diagnostic heuristics. Employing POCUS enables rapid, precise identification of reversible conditions, thereby providing valuable guidance for ongoing resuscitation efforts. In fact, the presence of return of spontaneous cardiac movement on ultrasound is strongly associated with return of spontaneous circulation and survival (1).

This chapter aims to improve the technique for performing POCUS and discuss its utility in saving lives.

## Objectives

1. Importance of performing POCUS

- Diagnostic clues from POCUS in cardiac arrest
- Monitoring and guidance of advanced resuscitation, with role in the termination of CPR

1. Performing POCUS during cardiac arrest

- Utility of probes
- Obtaining useful views

1. Timeline of performing POCUS during ACLS

## Importance of performing POCUS

Approximately 80% of in-hospital cardiac arrests are rhythms that are not amenable to shock, including pulseless electrical activity (PEA), pseudo-PEA, and asystole (2).

PEA specifically denotes the presence of organized electrical activity without effective cardiac contractions (cardiac standstill). Pseudo-PEA highlights a deceptive scenario where the observed electrical activity on the ECG does not correlate with an actual palpable pulse (despite cardiac contraction) (3). Asystole is a state of complete cessation of both electrical activity and contraction. Patients in PEA have hospital discharge survival rates ranging from 0.0 to 0.6%, whereas pseudo-PEA has been linked to a higher probability of achieving return of spontaneous circulation (ROSC). About 30% of in-hospital cardiac arrests are asystole. However, a study by Breitkreutz et al. (4) demonstrated that asystole documented on ECG alone could be misleading, and up to 35% of patients reported to be in asystole demonstrated coordinated cardiac motion on echo. Hence, ultrasound detection of cardiac activity might change the cardiac arrest algorithm by relying on real-time visualization of the heart rather than on rhythm analysis to assume cardiac standstill. However, this is still in its infancy, and the 2025 AHA ACLS guidelines recommend that experienced professionals use point-of-care ultrasonography (POCUS) during cardiac arrest to diagnose reversible causes, provided it can be performed without interrupting resuscitative efforts (i.e., CPR) (5).

### Diagnostic clues from POCUS in cardiac arrest

Traditionally, ACLS had categorized reversible causes of cardiac arrest into H’s and T’s (Hypoxia, Hypovolemia, Hyperkalemia, Hypothermia, Thrombosis, tamponade, Tension pneumothorax, Toxic agents). Usually, history, physical exam and pertinent labs assist in excluding potential etiologies. However, using POCUS can help us narrow down our differential diagnosis in the event of a cardiac arrest. Among the reversible causes, pocus might help diagnose or rule out cardiac tamponade, tension pneumothorax, and thrombosis (massive PE).

### Cardiac tamponade

**Figure 1:**
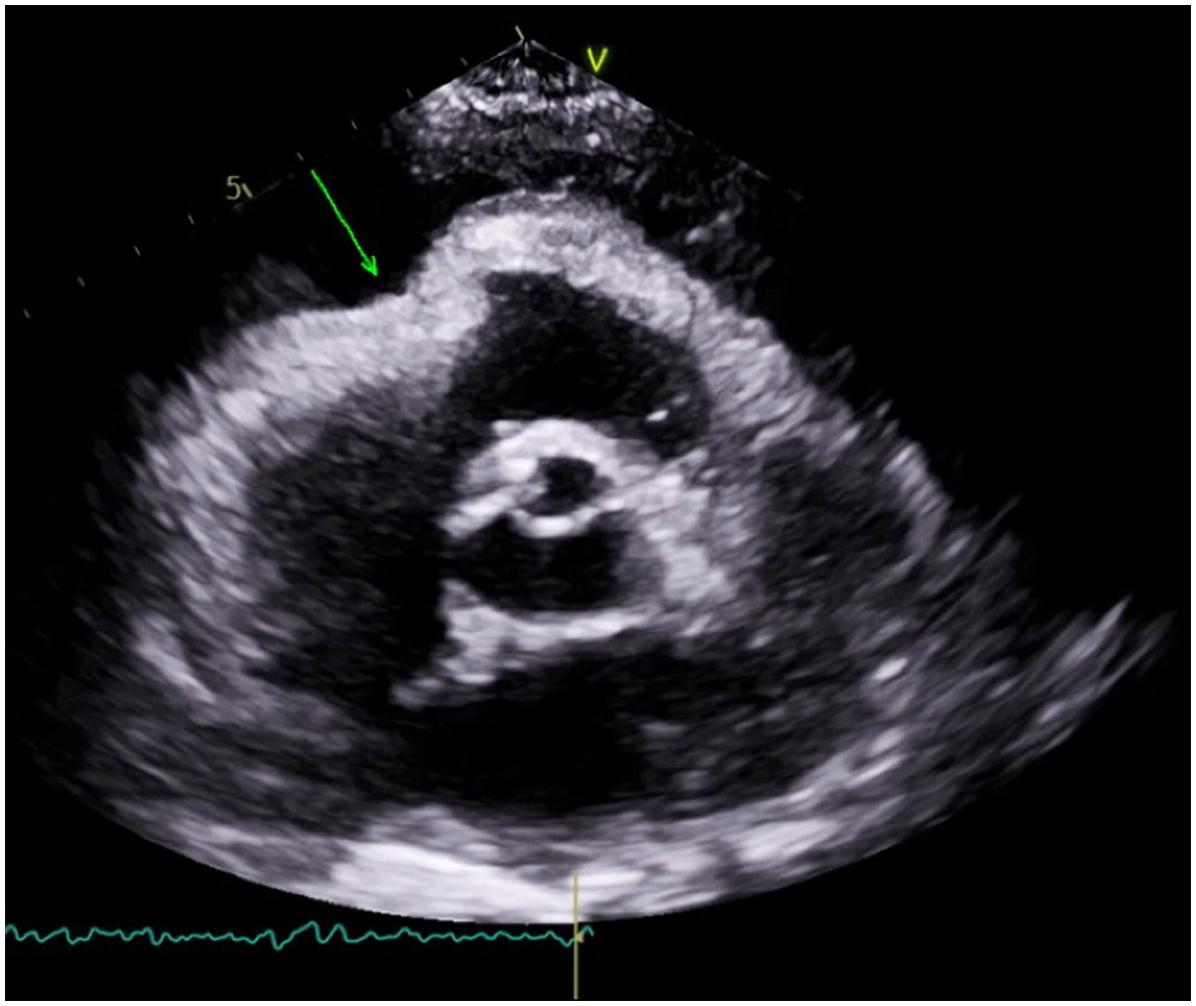
Parasternal short axis view showing pericardial effusion with RV collapse.

Ultrasonographic signs consistent with tamponade include the presence of fluid between the fibrous and serous pericardium, the collapse of the right cardiac chambers during diastole, and a small ventricular size (Figure 1). A dilated and poorly collapsible inferior vena cava may be observed as well. In the presence of suspected or confirmed cardiac tamponade with suspicion for interventricular dependence, an emergency pericardiocentesis to drain the accumulated fluid is indicated to restore circulation. Patients with pericardial effusion and pericardiocentesis had higher survival rates (15.4%) than those without (1.3%) (6).

### Thrombosis

A massive pulmonary embolism can cause cardiac arrest, resulting in PEA. The identification of PEA in this case can change management, including the potential use of thrombolytic therapy during cardiac arrest.

1. Dilated right ventricle with McConnell’s sign with a small LV cavity-The diagnosis of dilation relies on a comparison between the diameters of the RV and LV at the end of diastole. A normal relationship is defined as 0.6:1 and can be evaluated via an apical four-chamber view, while a ratio of 1:1 is diagnostic of RV dilatation. However, the relationship between RV dilatation and PE can be weak; in patients with cardiac arrest, RV dilatation can develop over the duration of the arrest. McConnell’s sign refers to akinesis of the RV free wall with preserved or hyperdynamic contraction of the RV apex. In a massive pulmonary embolism, the RV suddenly faces a wall of pressure and dilates like an overinflated balloon. This swollen RV shoves the interventricular septum toward the LV. At the same time, LV preload falls sharply because almost no blood is making it across the lungs. The LV ends up small, starved, and barely filling. Cardiac output plummets in a downward spiral. Clinically, the patient may lose a palpable pulse, yet the EKG still shows an organized rhythm. This is pulseless electrical activity, but if you place an ultrasound probe and spot even faint cardiac contractions, it is actually a pseudo-PEA: electrical activity with mechanical activity too weak to generate a pulse, which has a better outcome. In this patient, emergent thrombolytic therapy during cardiac arrest might help achieve ROSC.
2. Bulging of IVS to the left and a D-shaped RV-the right ventricle can present in a D shape, as visualized in a parasternal short-axis view.

### Tension pneumothorax

Pneumothorax is a clinical diagnosis with the patient presenting with sudden onset chest pain, shortness of breath, hypotension, tachycardia, and hyperresonance to percussion of the affected side of the lung. Given the history’s vagueness and, in some cases, its unavailability in this cohort of patients, our diagnostic heuristic is handicapped. A quick point-of-care ultrasound can effectively rule out a tension pneumothorax, with some limitations.

Ultrasonographic evidence of pneumothorax

1. Absence of lung sliding – Normal lung sliding implies the visualization of shimmering, synchronized movement of the visceral and parietal pleura during respiration. This phenomenon indicates that the two pleural layers are in direct contact and slide freely over each other with each breath. If air in the pleural space separates the parietal and visceral pleura, it prevents their normal movement, resulting in the loss or absence of lung sliding.

However, caveats to this include: absence may be seen in pleural adhesions, decreased ventilation (including apnea), lung collapse, unilateral lung intubation, and lung sliding only; these rule out the absence of a pneumothorax at the site of exam. However, for the purpose of ruling out tension pneumothorax in cardiac arrest, its presence is sufficient to do so.

**Figure 2:**
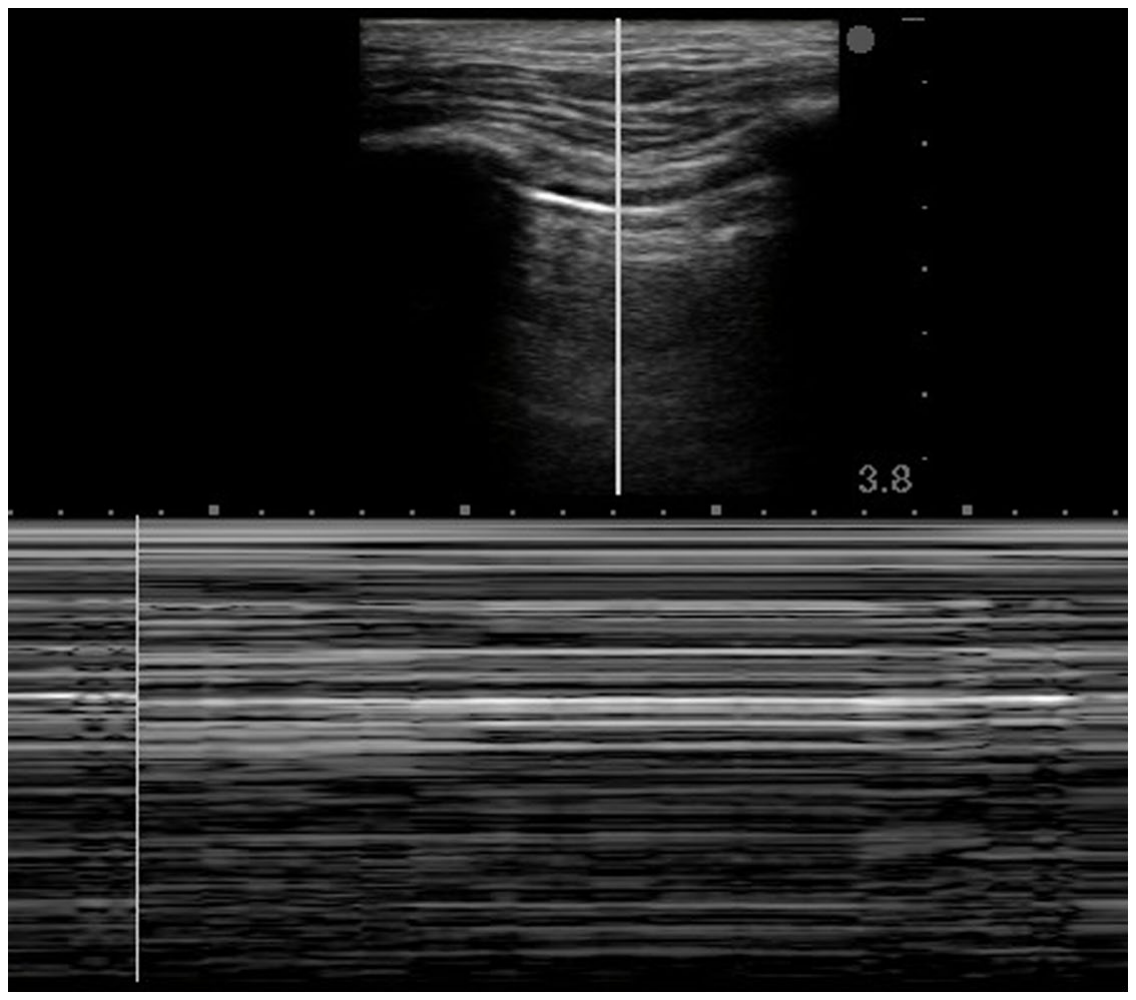
Barcode sign-M Mode demonstrating loss of pleural motion suggestive of pneumothorax.

1. Lung point – The interface of where the healthy lung starts and where the pneumothorax ends is known as the lung point. On one side of the lung point, healthy pleura will be seen with pleural sliding, whereas on the other, the pneumothorax will show a still pleural line with absent sliding. Visualization of the lung point is diagnostic of pneumothorax.
2. Utility of M Mode – Sea Shore and Barcode sign (Figure 2).

On gray-scale M-mode ultrasound, classical signs are:

- Seashore sign: normal lung sliding
- Barcode/stratosphere sign: pneumothorax
- Presence of B lines – B-lines in the lung are vertical, hyperechoic artifacts that appear on a lung ultrasound and originate from the pleural line, extending to the bottom of the screen without decreasing in intensity. Presence of B lines indicates absence of pneumothorax

### Monitoring and guidance of advanced resuscitation, with a role in the termination of CPR

In a prospective study by Heydari et al. (7) of 154 adults with non-shockable cardiac arrest, 24.7% achieved ROSC, and 5.8% survived to discharge. Initial cardiac activity on ultrasound was strongly predictive: patients with activity had a 52% ROSC rate compared with 12% in those without. The duration of cardiac standstill was the key prognostic marker, averaging 2.8 min in patients with ROSC versus 19 min in those without ROSC. Notably, no patient with ≥10 min of persistent standstill achieved ROSC, and no patient with ≥6 min of standstill survived to discharge. The authors conclude that serial POCUS during CPR can reliably identify when resuscitation is futile, though decisions should still incorporate overall clinical context rather than ultrasound alone (7)—however, an attempt at a meta-analysis by Zaki et al. (8) realized that there was a wide variation in the definition of cardiac activity, and the statistical heterogeneity was high; hence, they were unable to carry out meta-analyses. Instead, they concluded that POCUS has the potential to provide valuable information on the management of cardiac arrest patients; however, it should not be used as the sole predictor for the termination of resuscitation efforts (8).

In addition to prognostication, POCUS plays an important role in real-time detection of return of spontaneous circulation (ROSC). The reappearance of coordinated cardiac activity on ultrasound may precede the return of a palpable pulse or measurable blood pressure, particularly in cases of low-flow states. Recent work has emphasized that ultrasound can assist in identifying ROSC even during ongoing chest compressions, helping clinicians distinguish true PEA from pseudo-PEA and guiding immediate management decisions. Ultrasound-based POCUS carotid artery compression (POCUS-CAC) is a rapid method to detect return of spontaneous circulation (ROSC) during CPR by placing a linear probe transversely on the lateral neck, visualizing the carotid artery and internal jugular vein, and applying probe pressure: full carotid collapse with no pulsation suggests no ROSC, while resistance to compression/partial collapse or visible pulsation suggests ROSC (9). In a prospective ED study of 41 cardiac arrest patients (1984 POCUS-CAC checks vs. 496 manual pulse checks), POCUS-CAC identified pulses faster (mean 2.3 s vs. 4.7 s with manual palpation), detected ~53% of ROSC cases earlier (often during ongoing compressions), and showed high diagnostic performance (reported sensitivity 100%, specificity 87.5%), suggesting it can reduce pulse-check interruptions and support earlier ROSC recognition in resuscitation (9). Importantly, ultrasound assessment for ROSC must be integrated into structured pulse checks and should not prolong interruptions in chest compressions. When used appropriately, POCUS enhances situational awareness and supports timely clinical decision-making during resuscitation.

## Performing POCUS during cardiac arrest

### Utility of probes and obtaining useful views

The key to performing pocus during cardiac arrest is not to interrupt the arrest itself and to minimize the pause. POCUS should be performed within the 10-s pulse-check window. The optimal window for cardiac POCUS during CPR depends on the patient’s body habitus, patient positioning, and suspected underlying etiology. The subxiphoid view, also known as the subcostal view, is the default POCUS view in cardiac arrest because it does not interfere with CPR. However, in a multicenter RCT of 6,247 ultrasound images, the parasternal long-axis (PLAX) view achieved faster acquisition (8.8 s vs. 9.3 s) and higher high-quality imaging rates (66% vs. 58%) compared with the subxiphoid view (SubX) during brief emergency echocardiography (10).

#### Ultrasound pause-minimizing tips

- Pre-pause imaging can save about 15 s in pre−/post-implementation protocols.
- Limit clip length to 6 s or less, and save only one clip.
- Do not interpret in real time, resume compressions immediately and review later.
- The team leader should not perform the ultrasound.
- Use a real timer to track pauses.
- Assign the most experienced sonographer available.
- Follow a structured protocol such as CASA Protocol (Cardiac Arrest Sonographic Assessment) - A standardized ultrasound protocol designed for cardiac arrest to help clinicians rapidly detect reversible causes without prolonging CPR pauses (11).

### Timeline of performing POCUS during ACLS

#### How CASA works

##### Pre-pause preparation

Before the rhythm check:

- Position the ultrasound machine next to the patient
- Select the appropriate probe (usually the cardiac probe)
- Probe on the chest, but not to start scanning yet
- Select your preset and adjust device settings (depth, gain) before interruption
- Plan the target view (usually subxiphoid or parasternal long axis)
- Ensure recording is ready
- Assign roles so that the ultrasound operator is not the team leader

This prep prevents fumbling during the actual pause.

##### During the rhythm check (maximum 10 s)

- Clearly announce “Pause for ultrasound in 3, 2, 1”
- The designated sonographer (not the team leader) places the probe in the subxiphoid position during ongoing compressions to optimize position before the pause
- CPR is paused only for rhythm and pulse check
- Acquire one short clip (≤6 s)
- The timekeeper counts aloud to ensure the pause does not exceed 10 s. This task can be performed by the team leader
- Announce “Resume compressions in 3, 2, 1”
- CPR is resumed immediately after clip acquisition
- Image interpretation is completed only after compressions have restarted

##### Post-pause review

- Once compressions restart
- Review the saved clip while CPR continues

In practice, image acquisition should occur within the pulse-check window, while interpretation should occur during ongoing CPR. This division of tasks—acquisition during pause, interpretation during compressions—helps ensure that ultrasound enhances rather than delays high-quality resuscitation.

CASA proposes a sequential approach to evaluate different aspects without prolonging CPR interruption:

- First cycle (0–10 s): Subxiphoid view to look for cardiac tamponade.
- Second cycle (0–10 s): Parasternal long axis view to evaluate ventricular function and look for right ventricular dilation.
- Third cycle (0–10 s): Assess for cardiac activity.
- Fourth cycle (0–10 s): Rapid pulmonary assessment to look for pneumothorax.

The emphasis is on performing a single assessment per CPR cycle, prioritizing the most critical information.

#### What CASA prevents

- Long pauses in compressions
- Repeated or prolonged scanning
- Real-time interpretation delays
- Disorganized team workflow
- Team leader distracted by scanning

The biggest challenge in doing bedside POCUS during cardiac arrest is to avoid interrupting CPR for the purpose of pocus. Thus, point-of-care cardiac imaging must be performed during the pulse-check intervals, which last less than 10 s. Thus, every second counts. It requires a trained physician to perform the examination and the presence of a timekeeper who counts aloud during pulse checks.

### Limitations

Training is crucial for effectively performing CASA in 10 s. It should include practicing CASA in simulated cardiac arrest scenarios, with strict timing, regular training to obtain key ultrasound views quickly, and training the entire resuscitation team to integrate CASA without disrupting CPR. However, only a small number of observational studies, with significant limitations, including variability in the definitions of sonographic findings, describe the use of POCUS in this setting.

POCUS findings during cardiac arrest should always be interpreted within the broader clinical context rather than used as independent decision-making tools. The 2025 American Heart Association guidelines stress that POCUS must be performed with careful attention to minimizing interruptions in CPR and highlight that current evidence shows no improvement in outcomes when it is used during cardiac arrest (12). For example, right ventricular dilation observed during arrest is not specific for pulmonary embolism; experimental data demonstrate that right ventricular enlargement occurs across multiple arrest etiologies—including pulmonary embolism, hypoxia, and primary arrhythmia—with only modest diagnostic accuracy among physicians (13). Similarly, the presence of cardiac motion on ultrasound does not ensure sustained return of spontaneous circulation (ROSC) (14, 15). Systematic reviews indicate that no single sonographic finding has adequate sensitivity or consistency to justify terminating resuscitation efforts, and subtle findings such as isolated valve fluttering or minor myocardial twitching may not represent a perfusing rhythm (14, 15). Future research that standardizes image acquisition and other study methodology to examine the utility of POCUS for this indication is required.
